# Coexistence of myasthenia gravis and lichen planus: A case report and systematic review of related case reports from 1971 to 2024

**DOI:** 10.1002/ccr3.9065

**Published:** 2024-06-14

**Authors:** Melika Jameie, Mobina Amanollahi, Bahareh Ahli, Ghasem Farahmand, Hana Magrouni, Payam Sarraf

**Affiliations:** ^1^ Iranian Center of Neurological Research, Neuroscience Institute Tehran University of Medical Sciences Tehran Iran; ^2^ School of Medicine Tehran University of Medical Sciences Tehran Iran; ^3^ Neurology Department, Imam Khomeini Hospital Complex Tehran University of Medical Sciences Tehran Iran

**Keywords:** autoimmune diseases, lichen planus, myasthenia gravis, paraneoplastic syndromes, thymoma

## Abstract

**Key Clinical Message:**

The co‐occurrence of myasthenia gravis (MG) and lichen planus (LP) is a rare phenomenon, with only 13 cases reported in the English literature between 1971 and 2024. Patients with MG or LP, regardless of the thymoma status, require close monitoring for other autoimmune diseases.

**Abstract:**

Myasthenia gravis (MG) is an uncommon autoimmune disease, resulting in fatigable muscle weakness in the ocular, bulbar, and respiratory muscles, as well as muscles of the extremities. Lichen planus (LP) is an autoimmune mucocutaneous disease, presenting with pruritic and violaceous plaques on the skin and mucosal surfaces. So far, MG and LP co‐occurrence is only reported in anecdotal individuals. This study reports a patient with MG and LP and systematically reviews the English literature on this rare co‐occurrence from 1971 to 2024, indicating only 13 cases with similar conditions. A 67‐year‐old man presented with ocular and progressive bulbar symptoms, a year after being diagnosed with generalized LP. Laboratory evaluations were normal except for the high anti‐AchR‐Ab titer and a positive ANA titer. Neurologic examinations revealed asymmetric bilateral ptosis, weakness and fatigability in proximal muscles, and a severe reduction in the gag reflex. He was diagnosed with late‐onset, seropositive MG. The treatment included pyridostigmine (60 mg, three times daily), intravenous immunoglobulin (25 g daily for 5 days), and oral prednisolone. There was no evidence of thymoma in the chest x‐ray and CT scan without contrast. However, a CT scan with contrast was not performed due to the patient's unstable condition. A common autoimmune mechanism may underlie the unclear pathophysiology of MG and LP co‐occurrence, with or without thymoma. Patients with MG, LP, or thymoma require close monitoring and assessment for other possible autoimmune diseases.

## 1 BACKGROUND

Myasthenia gravis (MG), is an autoimmune neurological disorder caused by autoantibodies attachment to nicotinic acetylcholine receptors (nAChR) at the neuromuscular junction (NMJ) of skeletal muscles.^1^ This rare disorder has an incidence of 2.1 to 5.0 per million people per year and a prevalence of 7 to 20 per 100,000 people.^2^ Fatigable weakness is the clinical hallmark of this disease, defined as fluctuating muscle weakness that exacerbates with activity and alleviates with rest.^1^ Ocular symptoms, including ptosis, diplopia, photophobia, and blurry vision are also common features.^3^ Additionally, bulbar muscles can be involved in the course of MG, resulting in dysphagia, dysarthria, and dysphonia.^1^

MG can be categorized according to its clinical manifestations, age at onset, and type of autoantibodies. There are five main categories for MG based on the clinical manifestations.^4^ Class I is defined as limited ocular involvement in the absence of other symptoms^4^; class II, III, and IV are characterized by mild, moderate, and severe weakness affecting other than ocular muscles, with or without ocular muscle weakness of any severity,^4^ and class V is characterized by the need for intubation.^4^ Classes II, III, and IV are further classified into “a” and “b” subgroups: subgroup “a” describes a predominant weakness in voluntary muscles (limbs or axial muscles), and subgroup “b” describes predominant oropharyngeal or respiratory impairment.^4^ Considering the age of onset, MG is categorized as early‐onset (< 50 years) and late‐onset (≥ 50 years).^5^ According to the types of autoantibodies (Ab), patients are classified into seropositive (positive for anti‐acetylcholine receptor antibody [anti‐AchR Ab]) and seronegative (negative for anti‐AchR Ab),^6^ accounting for 80% and 20% of patients with MG, respectively.^2^ Autoantibodies against muscle‐specific tyrosine kinase protein (Anti‐MuSK‐Ab) are produced by 20%–50% of seronegative patients, resulting in MuSK‐associated MG.^1^, ^2^, ^5^ Other subgroups, including lipoprotein‐related protein 4 (LRP4)‐associated MG (autoantibody against LRP4) and antibody‐negative generalized MG (without detectable AChR, MuSK, or LRP4 antibodies) are also reported.^5^, ^7^

Early in the presentation of possible MG, the focus of the differential diagnostic considerations primarily hinges on the initial signs and symptoms.^5^, ^8^, ^9^ Conditions that may mimic ocular myasthenia encompass thyroid ophthalmopathy, chronic progressive external ophthalmoplegia, myotonic dystrophy, and oculopharyngeal dystrophy, as well as brainstem and motor cranial nerve pathology.^5^, ^8^, ^9^ Bulbar symptoms of MG may resemble manifestations of motor neuron disease, obstructive lesions of the oropharynx, brainstem disorders (i.e., gliomas), or multiple cranial nerve palsies.^5^, ^8^, ^9^ Diagnostic considerations for limb weakness symptoms associated with MG encompass motor neuron disease, chronic inflammatory demyelinating polyneuropathy, other motor neuropathies, Lambert‐Eaton myasthenic syndrome (LEMS), and myopathies.^5^, ^8^, ^9^ While isolated respiratory involvement is uncommon in MG, it may also manifest in motor neuron disease and acid maltase deficiency myopathy.^5^, ^8^, ^9^ Lastly, several conditions may mimic generalized myasthenia, including generalized fatigue, motor neuron disease, and LEMS.^5^, ^8^, ^9^ Other differential diagnoses include cerebral venous thrombosis,^10^ multiple sclerosis,^9^ and posterior reversible encephalopathy syndrome.^11^, ^12^

MG treatment options fall into four main categories: symptomatic treatment (anticholinesterase agents), chronic and rapid immunomodulating therapies, and surgery (thymectomy).^13^ The choice of therapy depends on the severity and progression of the disease, as well as the time it takes for each therapy to show clinical effect.^13^ Pyridostigmine bromide is the primary medication used for symptomatic treatment.^13^ However, most patients eventually require immunotherapy. Immunomodulating therapies aim to control the underlying immune response in MG.^13^ Chronic immunomodulating therapies, including glucocorticoids, azathioprine, cyclosporine, mycophenolate, and so forth, offer long‐term benefits.^13^ Rapid immunomodulating therapies, including plasmapheresis and intravenous immune globulin (IVIG), provide faster relief but are typically used in specific situations, such as myasthenic crisis, before thymectomy, as a bridge to slower‐acting treatments, or as an adjunct to other medications in refractory cases.^13^ Thymectomy is particularly beneficial for patients with nonthymomatous generalized seropositive MG, but its effects take months to years to develop.^13^

LP is a rare idiopathic autoimmune inflammatory skin disease, affecting 0.5 to 1% of the population.^14^ Although it may occur at any age, most patients present in the third or sixth decade of life.^15^ Classic LP is presented as “6 Ps” (planar, purple, polygonal, pruritic, papules, and plaques) mostly on the flexural surfaces of the extremities.^16^ LP could also affect other parts of the body, including cutaneous tissues other than skin (scalp, hair, nails) and mucus membranes (genitalia, esophagus, conjunctiva).^14^, ^17^ Therefore, several variants are defined for LP, including oral, nail, linear, vulvovaginal, annular, atrophic, ulcerative, and generalized LP.^14^ Notably, generalized LP has seldom been reported in the literature;^14^ it is characterized by rapidly spreading and disseminated lesions on the trunk, extremities, and mucous membranes.^18^, ^19^ LP might be found with other conditions of impaired immunity, including thymoma,^20^, ^21^, ^22^, ^23^, ^24^, ^25^, ^26^, ^27^, ^28^ ulcerative colitis (UC),^29^, ^30^ alopecia areata, vitiligo, and other skin disorders.^23^, ^24^, ^30^ Furthermore, it has been shown to be correlated with hepatitis C virus (HCV) infection.^31^, ^32^

In the present study, we discuss a rare coincidence of MG and LP in an elderly patient who had been diagnosed with generalized LP followed by the development of MG symptoms almost a year later. The coexistence of MG and LP is a unique scenario, as there are anecdotal reports of such cases in the existing literature.^22^, ^23^, ^25^, ^27^, ^29^, ^33^, ^34^, ^35^, ^36^ We have also conducted a systematic review of similar reports in the literature up until February 2024.

## 2 METHOD

### 2.1 Objectives and review questions

There were two objectives of this study.

1. Reporting a patient in our center who developed MG and LP within a year.
2. Systematically reviewing the literature for studies addressing the co‐occurrence of MG and LP.

### 2.2 Study design, information sources, and search strategy

This study was conducted at an academic hospital complex affiliated with Tehran University of Medical Sciences (TUMS), Tehran, Iran, and approved by the ethics committee of the university. As the patient was deceased, written informed consent for participation and publication of this manuscript was obtained from the patient's next of kin. The case study is reported according to the Consensus‐based Clinical Case Reporting Guideline Development (CARE) guidelines (Table S1).

For the second objective, we performed a comprehensive search for publications up till *February 01*, *2024*, through MEDLINE (via PubMed), Scopus, and Web of Science database, using the following medical subject headings (MeSH) and/or Title/Abstract/Keywords: “myasthenia gravis,” “myasthenia,” “myasthenic,” “lichen planus,” “lichenoid.” The detailed search strategy is presented in Table S2. Additional sources were identified through cross‐referencing.

### 2.3 Eligibility criteria

All original studies written in English that explored the coexistence of confirmed diagnoses of MG and LP were eligible to be included, regardless of the presence of other diseases. Review articles, editorials, clinical guidelines, studies that reported patients with either MG or LP (but not both), and any articles that did not align with our research question were excluded. No prior restrictions were imposed on study design (except for review articles, editorials, and guidelines), country, publication year, participant age, or any other factors.

### 2.4 Selection process

Initially, data records were imported into the EndNote software (version X9, Clarivate Plc) from various databases. Following the removal of duplicates, two separate researchers (M.J. and M.A.) reviewed all the records based on their titles and abstracts. The full texts of records that seemed potentially eligible were then evaluated further by three independent researchers (M.J., M.A., and B.A.). Any records that didn't meet the predefined eligibility criteria were excluded. Disagreements were settled by achieving a consensus.

### 2.5 Data collection

The following data were extracted for each study: study‐related variables (authors and published year), population‐related variables (age, sex, past medical history [PMH], and other immune‐related comorbidities), as well as MG‐ and LP‐related variables (symptoms and signs, age at diagnosis, treatment, and outcome).

## 3 RESULTS

### 3.1 Case presentation

A 67‐year‐old man, who was diagnosed with LP1 year ago, was admitted to the hospital whose symptoms had initiated 20 days before. The earliest symptom was mild bilateral ptosis. Progressive dysphagia and dysarthria were gradually added during the disease. In the beginning, the patient suffered from dysphagia to solids which progressed to dysphagia to liquids 3 days before attending the hospital. He developed dysarthria almost simultaneously with dysphagia in the form of nasal speech and unintelligible words, worsening with prolonged talking. However, his ability to understand was intact. The patient reported that his symptoms were milder in the morning and exacerbated during the afternoon. He did not report any difficulty in breathing. As well, he did not mention any other associated symptoms, such as headaches, diplopia, blurred vision, vertigo, dizziness, ataxia, or gastrointestinal symptoms. His medical history included primary hypertension, diabetes mellitus type 2, as well as multiple rapidly spreading purplish and pruritic lesions, which had been diagnosed as generalized LP a year before. There was no history of recent travel or infection or any particular food intake. Family and habitual history were unrevealing.

The initial physical examination showed an elderly, alert, and oriented male, without any signs of respiratory distress. He answered the questions cooperatively, albeit incomprehensible due to dysarthria. Vital signs were overall stable: *T* = 37.1°^C^, BP = 124/85 mmHg, HR = 87 beats per minute, RR = 14 breaths per minute, and O_2_ saturation in the room air = 98%. Flat‐topped, polygonal, violaceus papules were found all over the skin. Additionally, hyperpigmented macules and cutaneous eruptions were evident, particularly on the flexural surfaces of the lower extremities. There were no signs of mucous membranes, genitalia, nails, hair, or scalp involvement. Neurologic examinations revealed asymmetric bilateral fatigable ptosis with no evidence of ophthalmoplegia. There were no signs of facial asymmetry or sensory deficit. The gag reflex was severely decreased. The uvula was not deviated, and the soft palate elevation was symmetric. No signs of tongue deviation or fasciculation were present. The motor examination revealed fatigability and weakness in the proximal muscles of the upper and lower extremities (Table 1). Deep tendon reflexes (DTR), the superficial abdominal reflex, and plantar reflexes on both sides were normal. The remainder of the physical examination, including sensory‐neural and cerebellar examinations, did not disclose any other abnormalities.

**TABLE 1:** Patient history taking and physical examination results.

History taking and general physical examination	
History of multiple rapidly spreading purplish and pruritic lesions all over his body, more noticeable in his legs, beginning a year ago Mild bilateral ptosis better in the morning and exacerbated during the afternoon Progressive dysphagia for solids and liquids Progressive dysarthria (nasal speech and unintelligible words) worsening with prolonged talking Vital signs: stable Disseminated, violaceous, flat‐topped, and polygonal skin papules, hyperpigmented macules, and cutaneous eruptions were evident, particularly on the flexural surfaces of the lower extremities	
Neurologic examination	
*Mental status*: normal	
*Cranial nerves* Asymmetric bilateral ptosis with fatigability with no evidence of ophthalmoplegia Other neurological assessments contributed to the optic, oculomotor, trochlear, and abducens nerves were normal No signs of facial asymmetry or facial sensory deficit The gag reflex was severely decreased The uvula was not deviated, and the soft palate elevation was symmetric No signs of tongue deviation or fasciculation were present	
*Motor examination* ^a^	
Upper extremities	
Proximal	Tone: 5/5; Force: 5/5 with fatigability
Distal	Tone: 5/5; Force: 5/5
Lower extremities	
Proximal	Tone: 5/5; Force: 5/5 with fatigability
Distal	Tone: 5/5; Force: 5/5
Head	Tone: 5/5; Force: flexion:4/5, extension: 5/5
Trapezius	Tone: 5/5; Force: 5/5
*Sensory examination*: Normal	
*Coordination*: Normal	
*Reflexes*: Normal	
*Gait*: Normal	
Imaging studies	
CXR	Normal
Chest CT scan (without contrast)	Normal
MRI of the brain and orbit	Normal
Electrodiagnostic studies	
Motor and sensory NCS	Normal
CMAP	Normal
Needle EMG of distal and proximal muscles	Normal
RNS	↓ Response in the anconeus, trapezius, and orbicularis oculi muscles

Abbreviations: CMAP, compound muscle action potentials; EMG, electromyography, NCS, nerve conduction studies, RNS, repetitive nerve stimulation.

^a^ Scoring is based on the Medical Research Council (MRC) scale.

An electrocardiogram (ECG) showed normal sinus rhythm. The initial patient's laboratory tests showed no abnormality (Table 2). In the following days, a comprehensive autoimmune workup was performed; rheumatologic laboratory tests and thyroid function tests were performed to rule out systematic lupus erythematosus (SLE), rheumatoid arthritis (RA), and Graves' disease. MG‐specific tests, including anti‐AchR and anti‐MuSK‐Abs, were performed, with a positive titer of 5.43 nmol/L for anti‐AchR‐Ab and negative results for anti‐MuSK‐Ab. The patient was diagnosed with late‐onset seropositive class “IVb” MG. The treatment included pyridostigmine (60 mg, three times daily) and IVIG (25 g daily for 5 days). Additionally, 50 mg oral prednisolone was initiated.

**TABLE 2:** Patient laboratory evaluations.

Initial tests	Value	Further tests	Value
Hg (g/dL)	13.6	IgA	302
WBC (10^3^/μL)	10.9	RF	58
PLT (10^3^/μL)	293,000	C3	97
PT (seconds)	12.2	C4	23
INR	1	ANA	1/320, Coarse Speckled
PTT (seconds)	25	Anti‐ds DNA	6.1
Glucose (mg/dL)	58	P‐ANCA	0.85
BUN (mg/dL)	1	C‐ANCA	1.80
Creatinine (mg/dL)	0.9	TSH (μIU/mL)	1.4
Sodium (mEq/L)	139	FT4 (μg/dL)	1.1
Potassium (mEq/L)	4.4	Anti‐AchR Ab	5.43
Calcium (mg/dL)	9.0	Anti‐MuSK Ab	Negative
Magnesium	1.9		
Phosphorus (mg/dL)	6.5		
AST (U/L)	22		
ALT (U/L)	25		
ALP (U/L)	112		

Abbreviations: AchR Ab, acetylcholine receptor antibody; ALP, alkaline phosphatase; ALT, alanine aminotransferase; ANA, antinuclear antibodies, anti‐ds DNA, anti‐double‐stranded DNA; anti‐MuSK Ab, anti‐muscle‐specific kinase antibody; AST, aspartate aminotransferase, BUN, blood urea nitrogen; C3, complement 3; C4, complement 4; C‐ANCA, cytoplasmic anti‐neutrophil cytoplasmic antibodies; FT4, free T4; Hg, hemoglobin, IgA: immunoglobulin A; INR, international normalized ratio; P‐ANCA, perinuclear anti‐neutrophil cytoplasmic antibodies; PLT, platelet; PT, prothrombin time; PTT, partial thromboplastin time; RF, rheumatoid factor; TSH, thyroid stimulating hormone; WBC, white blood cells.

Chest x‐ray (CXR) revealed no abnormality. Since a negative CXR does not rule out small thymomas, a chest CT scan was required.^1^ As the patient's condition worsened and dysphagia progressed, he was admitted to the intensive care unit (ICU). Due to the patient's unstable condition, contrast was not used for the chest CT scan, which showed no signs of thymoma or other abnormalities. MRI of the brain and orbit was unraveling. Routine motor and sensory nerve conduction studies (NCS), routine needle electromyography (EMG) of distal and proximal muscles, and compound muscle action potential (CMAP) revealed no abnormal findings. Repetitive nerve stimulation (RNS) of muscles at 3 Hz showed a decremental response in the anconeus, trapezius, and orbicularis oculi muscles, with reduced excitatory postsynaptic potentials (EPSPs) (Table 1).

### 3.2 Systematic literature search and study selection

**FIGURE 1:**
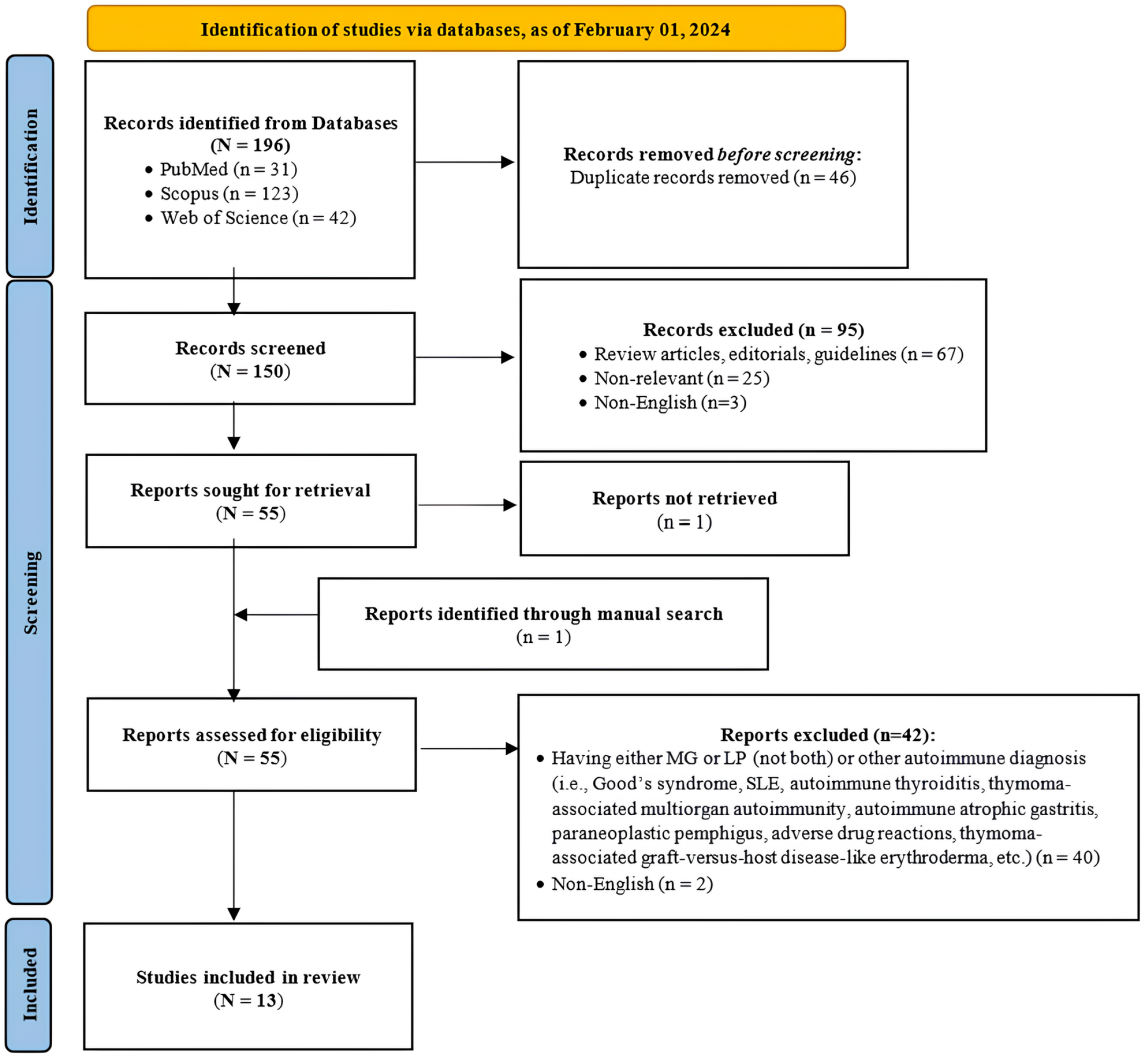
PRISMA 2020 flow diagram for new systematic reviews. From: Page MJ, et al.^37^

The PRISMA flowchart provides a visual representation of the inclusion process (Figure 1). Searching the databases resulted in 196 records. After duplication removal, 150 records were screened by titles and abstracts, of which 95 articles were excluded. Of the remaining 55 articles sought for retrieval, the full text of one article was not retrieved,^34^ and one additional study was found by manual search.^35^ Therefore, 55 studies were assessed for eligibility, 42 of which were excluded for the following reasons: having either MG or LP (not both) or other autoimmune diagnoses (i.e., Good's syndrome, SLE, autoimmune thyroiditis, thymoma‐associated multiorgan autoimmunity [TAMA], autoimmune atrophic gastritis, paraneoplastic pemphigus, adverse drug reactions, thymoma‐associated graft‐versus‐host disease [GVHD]‐like erythroderma, etc.) (*n* = 40), and non‐English articles (*n* = 2).^38^, ^39^ Eventually, 13 articles were included in this systematic review.

### 3.3 Study characteristics

Out of 14 patients with MG and LP co‐occurrence reported from 1971 to 2024 (including our patient and 13 from the literature), most were middle‐aged females (9/14). The MG diagnosis age ranged from 30 to 67 years, and the LP diagnosis age ranged from 26 to 67 years. In most of the patients, MG symptoms preceded LP.^20^, ^21^, ^27^, ^35^, ^40^ However, some patients developed LP before MG^36^ or experienced both disorders at the same age.^22^, ^23^, ^29^, ^30^, ^41^ The exact ages at MG diagnosis or LP diagnosis were not available for two patients.^42^, ^43^ Most of the patients (9/14) had a history of thymoma or were diagnosed with thymoma during hospitalization.^20^, ^21^, ^22^, ^23^, ^27^, ^35^, ^36^, ^42^, ^43^ However, evidence of thymoma was not found in 5/14 patients.^21^, ^29^, ^30^, ^40^, ^41^ Other comorbid autoimmune conditions in patients included Good's syndrome,^42^ T cell large granular lymphocytic leukemia,^42^ vitiligo,^23^, ^29^, ^30^, ^43^ pure red cell aplasia,^43^ SLE,^43^ pemphigus vulgaris,^43^ alopecia areata,^23^, ^29^, ^30^, ^43^ idiopathic thrombocytopenic purpura (ITP),^36^ and UC.^29^, ^30^

The treatment modalities used for MG included acetylcholinesterase inhibitors^21^, ^22^, ^23^, ^36^, ^41^ (pyridostigmine or neostigmine), IVIG (this study), plasmapheresis,^36^, ^40^ immunoadsorption,^40^ corticosteroids,^20^, ^21^, ^23^, ^29^, ^30^, ^35^, ^36^, ^40^ and thymectomy.^22^, ^36^ Treatment modalities for MG were not reported in three studies.^27^, ^42^, ^43^ The treatment modalities used for LP included topical immunosuppressants (corticosteroids^20^, ^22^, ^27^, ^40^, ^41^, ^42^ and topical tacrolimus^42^), systemic immunosuppressants (prednisone,^23^, ^27^, ^35^, ^36^, ^42^, ^43^ methotrexate,^42^ azathioprine^42^), acitretin,^40^, ^42^ phototherapy,^42^ and antihistamines.^42^ Treatment modalities for LP were not reported in three studies.^21^, ^29^, ^30^ A summary of the patients' characteristics is provided in Table 3.

**TABLE 3:** Cases reported in the literature with coexistence of MG and LP up till February 01, 2024.

Baseline demographic and clinical characteristics					Myasthenia gravis characteristics				Lichen planus characteristics			
Study	age	Sex	PMH^a^	Other autoimmune comorbidities	Age at Dx	Symptom/sign	Treatment	Outcome	Age at Dx	Symptom/sign	Treatment	Outcome
This study 2024	67	Male	HTN DM 2	There was no evidence of thymoma in chest CT without contrast No other immune comorbidity	67	Mild bilateral ptosis Progressive dysphagia and dysarthria (nasal speech and unintelligible words) No limb, axial, or respiratory muscle weakness	Pyridostigmine (60 mg, TID), IVIG (25 g daily for 5 days), oral prednisolone (50 mg)	Deceased	66	Multiple rapidly spreading purplish and pruritic lesions all over his body and more noticeable in his legs, beginning a year ago	NA	Relatively improved
Ho et al.^41^	67	Male	HTN	No evidence of autoantibodies and thymoma	67	Ocular presentation (fatigable ptosis of both eyelids with a positive Cogan's lid twitch sign, left head tilt, right eye hypertropia on the straightening of his head)	Pyridostigmine	Ptosis and head tilt showed improvement with pyridostigmine on subsequent follow‐up	67	Several hyperpigmented macules at several flexural and non‐sun exposed areas of the trunk (a 5 ×5 cm hyperpigmented, purplish patch with an irregular border at the center of the back + several hyperpigmented linear streaks in both axillae + two erythematous macules on chest)^b^	Topical betamethasone valerate 0.05% cream for 6 weeks	Subsequent visits did not show any new lesions or progression and the pigmentation remained stable
Bendayan et al.^42^	52	Female	NA	Malignant thymoma status post‐resection, recurrent pulmonary infections, candida gastritis, Good's syndrome, T cell large granular lymphocytic leukemia	NA	NA	NA	NA	52	Diffuse pruritic, hyperpigmented plaques with fibrosis	Topical steroids, topical tacrolimus (0.1%), topical glycolic acid (10%), oral methotrexate, azathioprine, acitretin, and narrowband UVB phototherapy, oral prednisone ‐For pruritus: hydroxyzine, cetirizine ‐Doxepin, naltrexone	Resistant to all of the therapies except for oral corticosteroids
Ge et al.^43^	64	Female	NA	MAS‐ Vitiligo, pure red cell aplasia, SLE, pemphigus vulgaris, alopecia areata, thymoma	49	NA	NA	NA	NA	Scattered erosive erythema with crusting and flaccid blisters on the scalp, trunk, and upper limbs, some irregular depigmented patches could be seen on the chest and face^c^	Methylprednisolone (24 mg/day) for MAS	Improved
Motegi et al.^27^	50	Male	NA	Thymoma	46	NA	NA	NA	50	Painful erosions on lips, tongue, and oral mucosa	Topical steroids, oral prednisolone	Improved
Alsenaid et al.^40^	65	Female	NA	A thymoma was excluded by radiology, autoimmune Diseases other than MG were excluded by clinical and laboratory testing including skin biopsies and direct immunofluorescence	30	A dull facial expression, weak hesitant speech, and generalized muscle weakness	Oral prednisolone (10 mg daily), plasmapheresis and immunoadsorption	NA	59	Generalized pruritic Skin eruption, generalized, symmetrical, Violaceous, lichenoid, flat‐topped papules and plaques, with fine transparent scales and Wickham signs, no mucous membrane involvement.	Resistant to topical therapy, successful treatment with systemic acitretin (20 mg daily)	improved
Qiao et al.^23^	53	Male	NA	After 7 months: scalp alopecia (diagnosed as alopecia areata), depigmented macules on trunk and hands (diagnosed as vitiligo)/a mass in the anterior mediastinum in CT scan (diagnosed as thymoma)	53	Progressive weakness, dysphonia, diplopia, dyspnea	Pyridostigmine (60 mg TID), Prednisone (30 mg daily)	Significant improvement in weakness	53	Oral and mucosal lesions; erosions on the tongue, buccal, and lips	Prednisone (30 mg daily)	no improvement in oral signs after medical therapy, oral lesions disappeared 1 month following the thymectomy
Giménez et al.^21^	60	Female	NA	Thymoma	54	Mild generalized MG	Anticholinesterase treatment	Good response	58	NA	NA	NA
	65	Female	NA	No evidence of thymoma	65	Ocular MG	Corticosteroids	Good response	67	NA	NA	NA
Mineo et al.^36^	32	Female	Splenectomy	Idiopathic thrombocytopenic purpura, thymoma	32	Weakness, shortness of breath, ptosis, myopathic facies, dysarthria, nasal speech dysphagia, gustatory and olfactory hallucinations, dysphoria	Before thymectomy: plasmaphereses, pyridostigmine bromide (60 mg/8 h orally) After thymectomy: prednisone (50 mg/day), pyridostigmine bromide (60 mg 8 h orally)	Improved myasthenic symptoms	NA (before MG)	Oral lesions	Steroids	improved after thymectomy
Helm et al.^35^	60	Female	NA	Metastatic adenocarcinoma of the right breast, a history of thymoma 7 years before	53	NA	Low dose prednisone	Treated	60	Long linear erosions on the tongue and the lower labial mucosa	Prednisone 16 mg daily, griseofulvin 250 mg BD	Treated, no recurrence
Pavithran et al.^22^	42	Female	NA	Thymoma	42	Fatigability, recurrent transient bilateral ptosis, dyspnea in the supine position, weakness of mastication	Before thymectomy: neostigmine bromide (15 mg orally four times a day)/after thymectomy: neostigmine bromide (30 mg orally in 2 doses)	Partial response to treatment before thymectomy/almost complete response to therapy after thymectomy	42	Pruritic, violaceous, and pigmented lesions on arms, legs, and chest, superficial erosions on the lips, gingiva, and tongue, white reticulated and erythematous lesions on the buccal mucosa	Topical corticosteroid ointments	Partial response
Aronson et al.^20^	54	Female	Open‐angle glaucoma, depression, cataract	Thymoma	53	Drooping of the mouth/bilateral ptosis/weakness of mastication	Oral prednisone (30 mg daily)	Total remission, without recurrence	54	Violaceous plaques on the extremities/erosive lesions in the vagina and mouth	Resistant to several topical therapies (topical steroids, and heparin sodium mouth rinses)	Only transient improvement after thymectomy (lasting a few weeks)
Tan et al.^30^ Miller et al.^29^	38	Male	Staphylococcal septicemia, pulmonary abscess, sinusitis	Ulcerative colitis, alopecia areata, vitiligo, splenomegaly No evidence of thymoma	35	Ocular presentation/striated muscle Three months later, a severe exacerbation of myasthenia occurred, requiring Artificial ventilation	IV edrophonium, then prednisone 10 mg daily	Excellent response to edrophonium, relatively controlled with prednisolone after exacerbation	26	Atypical lichen planus on the trunk presented with well‐circumscribed oval patches with slight central atrophy and lichenoid margin. confirmed by skin histopathology	NA	Exacerbated in 1968 (due to the treatment of sinusitis with penicillin)

Abbreviations: BD, twice a day; CT, computed tomography; IV, intravenous; IVIG, intravenous immunoglobulin; LP, lichen planus; MAS, multiple autoimmune syndrome; MG, myasthenia gravis; NA, not available; SL, systemic lupus erythematosus; TAMA, thymoma‐associated multiorgan autoimmunity; TID, three times a day.

^a^ A past medical history of diseases other than autoimmune conditions.

^b^ The patient was diagnosed with lichen planus pigmentosus, which is a variant of lichen planus.

^c^ The skin manifestations are related to both pemphigus vulgaris and lichen planus.

## 4 DISCUSSION

In this study, we described a patient with concomitant MG and LP and no evidence of thymoma. Previously, the association between MG and LP has been reported in a few cases. We reviewed a variety of patients based on the studies published between 1971 and 2024. Most patients had LP, MG, and thymoma together, but some only had LP and MG. Some patients also had other immune‐related disorders along with LP and MG, with or without thymoma.

### 4.1 Individuals with MG and LP co‐occurrence without thymoma

In 1971, Miller et al. reported a 35‐year‐old man who developed UC, followed by LP 4 years later. Nine years after LP diagnosis, he presented ocular symptoms of MG, and a period of severe relapse and respiratory failure occurred in the same year.^29^ In 1974, Tan et al. reported the subsequent events experienced by this patient; he developed alopecia areata a year after MG diagnosis, and vitiligo also appeared 3 years later.^30^ In 1997, Giménez‐Arnau et al. introduced two patients who experienced the coexistence of MG and LP.^21^ The first patient was a 60‐year‐old female with an incidentally diagnosed thymoma who developed oral LP and generalized seropositive MG, 1 year and 2 years after thymoma detection, respectively. The second patient was diagnosed with ocular MG at the age of 65, followed by developing LP lesions 2 years later. Further evaluations of this patient revealed no signs of thymoma.^21^ The authors mentioned two possible conclusions. First, the association of MG and LP in both cases could support their common autoimmune nature. Second, the presence of thymoma in the first case and the absence of that in the second patient could suggest that thymoma could not act as a primary event in MG.^21^ Consistently, in 2023, Ho et al. introduced a 67‐year‐old male presented with left head tilt associated with right eye hypertropia on the straightening of his head.^41^ Ophthalmology tests revealed fatigable ptosis of eyelids, as well as a positive Cogan's lid twitch sign. The patient was diagnosed with ocular MG and showed improvement with pyridostigmine. Three months later, he noticed a hyperpigmented, purplish patch at his back, along with several hyperpigmented streaks in both axillae and erythematous macules on the chest. The patient finally developed several hyperpigmented macules at the trunk and was diagnosed with lichen planus pigmentosus‐inversus. Topical betamethasone valerate was prescribed, and in subsequent follow‐up, the lesions didn't show progression and remained the same. There was no evidence of thymoma detected.^41^

### 4.2 Individuals with MG and LP co‐occurrence with thymoma

Recent studies showed that MG could be a part of a paraneoplastic syndrome associated with thymoma.^44^, ^45^ In 1978, Aronson et al. reported a 54‐year‐old woman who developed MG followed by LP a year later.^20^ Shortly after diagnosis, she presented with severe nonproductive cough and bilateral rhonchi on examination, leading to a thymoma diagnosis. In 1986, Pavithran et al. described a 42‐year‐old woman who developed MG secondary to thymoma. Simultaneously, as the initial symptoms of MG appeared, LP lesions became evident.^22^ In 1994, Helm et al. introduced a 60‐year‐old woman with metastatic breast cancer who had had benign thymoma along with MG 7 years earlier, treated with surgical extirpation and low‐dose prednisone. Since then, the patient experienced an oral erosive disease, presenting with long linear erosions on the tongue as well as lower labial mucosa which was diagnosed as LP through biopsy. The symptoms were finally controlled with prednisone and griseofulvin.^35^

In 1996, Mineo et al. described a 32‐year‐old woman who underwent splenectomy due to ITP in 1988.^36^ She was also diagnosed with oral LP. In February 1995, she presented with petechia and MG symptoms. Her symptoms well responded to plasmapheresis, pyridostigmine bromide, and prednisone. She also showed psychiatric symptoms including gustatory and olfactory hallucinations, dysphoria, and recent memory loss. Of note, she was diagnosed with thymoma which was excised and did not show recurrence following the operation. Interestingly, her LP‐associated oral lesions disappeared 2 months after the operation; therefore, steroids were discontinued. Furthermore, psychiatric symptoms improved 4 months after surgery. In 2015, Motegi et al. evaluated 50 patients with LP referred to their clinic between 2004 and 2014, identifying thymoma in three (6%) individuals. Among them was a 50‐year‐old male with erosive oral LP, who had developed MG at 46, with thymoma detection following a year thereafter.^27^

### 4.3 MG and LP: Possible shared pathophysiological mechanisms

The simultaneous occurrence of MG and lichen planus LP suggests underlying shared pathophysiological mechanisms that could include disruptions in immune function (i.e., T cell dysfunction), genetic vulnerabilities, and environmental triggers (i.e., infectious diseases).^20^

The thymus gland plays a critical role in both disorders. It provides a vital environment for T‐cell development, essential for immune surveillance and the prevention of autoimmune reactions.^46^ The process of thymopoiesis ensures a full complement of peripheral naïve T cells capable of diverse pathogen recognition, as well as regulatory T cells capable of suppressing hyperactive immune responses and autoimmunity.^46^ While early life thymus activity is crucial, the maintenance of T cell balance and proper immunoregulation in adulthood predominantly occurs through peripheral mechanisms for homeostasis.^46^, ^47^, ^48^ This may explain why some patients with MG and LP exhibit thymic involvement, while others do not. While the specific mechanisms of peripheral T cell turnover (outside the thymus) remain unclear, recent studies suggest lymph nodes might act as a tissue reservoir for naïve and resting T cell maintenance.^47^, ^49^

While the exact genetic predispositions to MG and LP are not fully understood, researchers have identified several genetic variations that increase susceptibility to these conditions.^50^, ^51^, ^52^, ^53^, ^54^ For instance, variations within the human leukocyte antigen (HLA) region, also known as the major histocompatibility complex (MHC), are associated with a higher risk of developing MG.^50^, ^51^ Similarly, genetic variants in the IL‐12/23 and IL‐17 receptor genes have been linked to an increased risk of LP.^52^, ^53^ Interestingly, the cytotoxic T‐lymphocyte antigen 4 (CTLA‐4) gene appears to play a role in both MG and LP, further suggesting that these conditions may share common underlying mechanisms.^51^, ^54^, ^55^

The HLA genes are responsible for producing proteins that present antigens to T cells, triggering a T‐cell‐mediated immune response,^56^ which plays a key role in autoimmune conditions due to mechanisms such as molecular mimicry.^56^, ^57^, ^58^ It is suggested that molecular mimicry is one of the principal mechanisms that foreign antigens lead to autoimmunity.^57^, ^59^ This occurs when cross‐reactive epitopes are displayed via the MHC, leading to an immune response through T‐cell activation.^57^, ^59^ The resulting proinflammatory response, crucial for eliminating pathogens, may continue if there is a sequential or structural similarity between foreign and self‐antigens.^59^ On the other hand, the CTLA‐4 is a co‐inhibitory molecule that plays a critical role in dampening immune responses to avoid autoimmunity, by suppressing T‐cell activation, proliferation, and cytokine production.^60^ Therefore, CTLA‐4 deficiency or mutation can lead to autoimmune diseases.^60^ However, while these genetic variations may suggest overlapping genetic predispositions that contribute to autoimmunity, further research is required in this regard.

Environmental factors also play a role, with both conditions possibly being influenced by variables such as smoking,^61^, ^62^ socioeconomic factors,^61^, ^63^ stress,^64^ viral infections,^53^, ^64^ and exposure to certain chemicals or drugs.^53^, ^64^ Overall, a combination of immune system dysfunction, genetic predisposition, and environmental factors, determines an individual's overall susceptibility to these autoimmune conditions.

### 4.4 Thymoma and autoimmune disorders: Possible mechanisms and associations

MG occurs in 50% of patients with thymoma (varying from 7% to 54%^65^), and is called thymoma‐associated MG (TAMG) in these cases.^66^ Notably, a multicenter retrospective study found a poorer prognosis for patients with TAMG compared to non‐thymoma MG.^67^ On the other side, approximately 10%–20% of patients with MG develop thymoma.^28^, ^68^ According to previous studies, thymoma has the greatest rate of paraneoplastic syndromes among all human cancers.^65^ The term “paraneoplastic syndrome” refers to a group of rare diseases with multiple systemic manifestations caused by an impaired immune response due to an underlying malignancy.^69^ Indeed, T cells generated by the neoplastic thymoma tissue are likely to be responsible for the autoimmune paraneoplastic features.^70^ Consistently, in 2011, Qiao et al. reported a 53‐year‐old male with a 6‐month history of myasthenic symptoms and oral mucosal lesions, which were diagnosed as LP after a buccal biopsy.^23^ A CT scan showed an anterior mediastinal mass, indicative of thymoma. Seven months after initiating treatment, he presented with scalp alopecia and vitiligo. Notably, his oral lesions, unresponsive to medical treatment, improved significantly 1 month after thymectomy.^23^

This finding aligns with reports of other patients experiencing remarkable improvement in symptoms of MG^22^ or LP^36^ after thymectomy. Contrarily, while the patient reported by Aronson et al. experienced some initial improvement in the mucocutaneous lesions several weeks following thymectomy, the lesions became worsened and more widespread only after a few weeks.^20^ Nevertheless, current guidelines support thymectomy as a treatment for MG due to its potential for improved clinical outcomes and long‐term benefits.^13^ Studies have shown that thymectomy roughly doubles the chance of achieving medication‐free remission and increases the likelihood of becoming symptom‐free by about 50%.^13^ However, it's important to note that the full benefits of thymectomy may take several years to develop.^13^ Notably, the role of thymectomy in improving LP has not yet been established and requires further research.^27^, ^71^, ^72^

In 2021, Bendayan et al. reported a case presenting with generalized lichenoid plaques.^42^ The patient was previously diagnosed with MG, Good's syndrome, T‐cell large granular lymphocyte leukemia, and malignant thymoma which had been resected multiple times. Of note, biopsy results revealed infiltration of CD8^+^ and CD4^+^ lymphocytes (with dominance of CD8^+^ cells), dyskeratosis, extensive pigmentary incontinence, as well as thickened collagen bundles in the dermis and the subcutaneous layer. Based on clinical and paraclinical findings, chronic GVHD, LP, and morphea in the setting of TAMA were considered differential diagnoses. Finally, according to the presence of malignant thymoma in the patient's history, TAMA was favored over the other differential diagnoses.^42^ TAMA is a rare paraneoplastic disease that is characterized by the involvement of the gastrointestinal tract, thyroid, and liver, as well as skin.^71^, ^73^

The underlying pathophysiology of TAMA is supposed to be the thymoma's contribution to the disruption of the thymus function in developing central tolerance and negative selection.^42^ Consistently, according to a study by Offerhaus et al., patients with TAMA showed decreased expression of autoimmune regulator genes (*AIRE*) *which* are expressed by the thymus and contribute to the negative selection of T cells.^74^ Consistently, Hoffacker et al. detected elevated levels of CD8^+^ autoantigen‐specific T cells in the blood of patients with thymoma.^75^ Taken together, the above‐mentioned hypotheses could explain the association of thymoma with several paraneoplastic and autoimmune disorders such as MG, lichenoid skin lesions, hypogammaglobulinemia, pure red cell aplasia, and RA.^28^, ^42^, ^76^, ^77^

The co‐existence of MG and LP has been also reported in the context of multiple autoimmune syndrome (MAS),^43^ as exemplified by the case reported by Ge et al. This involved a 64‐year‐old female with MAS, who underwent thymectomy 22 years prior and was subsequently diagnosed with an array of autoimmune conditions, including pemphigus vulgaris, MG, vitiligo, pure red cell aplasia, systemic lupus erythematosus, LP, and alopecia areata.^43^

### 4.5 Limitations and strengths

Although a contrast‐enhanced chest CT is essential for thymoma evaluation, the patient's instability necessitated reliance on a non‐contrast chest CT. The robustness of this study is enhanced by an exhaustive review of the pertinent literature from its origin to the present.

## 5 CONCLUSIONS AND FURTHER DIRECTIONS

While the exact pathogenesis of MG, LP, and thymoma co‐occurrence remains elusive, it suggests the possibility of a shared autoimmune mechanism. Various theories have been suggested to explain the association between thymoma and autoimmune disorders or paraneoplastic syndromes due to T lymphocyte dysfunction, genetic predisposition, and environmental factors. Nonetheless, instances of MG and LP co‐occurring without thymoma suggest that these conditions might arise independently of paraneoplastic syndromes. This supports the notion that a dysregulated immune response could account for the simultaneous presence of multiple autoimmune diseases within an individual.

Several key questions remain regarding the coexistence of MG and LP. Future research should delve deeper into the role and mechanisms of peripheral T‐cell homeostasis, particularly in patients without thymoma. Understanding how the body maintains T‐cell balance outside the thymus could shed light on the shared pathological mechanisms between these conditions. Additionally, further investigation is needed to elucidate the specific genetic variations that increase susceptibility to MG and LP. Prospective studies are crucial to definitively determine the role of thymectomy in improving LP. Understanding how thymoma disrupts the thymus's ability to develop central tolerance and negative selection of T cells also requires further exploration. These areas of research hold promise for improving our understanding of MG and LP, both with and without thymoma involvement. Ultimately, this knowledge could lead to the development of enhanced diagnostic tools, including specific biomarkers for early detection of MG and LP. Additionally, it may inform more effective therapeutic strategies, encompassing both established and potentially novel treatment approaches for these autoimmune conditions.

In conclusion, patients with MG, LP, or thymoma require close follow‐up and evaluation for other potential disorders, particularly autoimmune diseases.

## AUTHOR CONTRIBUTIONS

**Melika Jameie:** Conceptualization; data curation; investigation; methodology; project administration; supervision; visualization; writing – original draft; writing – review and editing. **Mobina Amanollahi:** Conceptualization; methodology; writing – original draft. **Bahareh Ahli:** Conceptualization; methodology; writing – original draft. **Ghasem Farahmand:** Data curation; writing – original draft. **Hana Magrouni:** Data curation; writing – original draft. **Payam Sarraf:** Conceptualization; investigation; project administration; supervision; validation; writing – review and editing.

## FUNDING INFORMATION

The authors received no financial support for the research, authorship, and publication of this manuscript.

## CONFLICT OF INTEREST STATEMENT

The author has no conflicts of interest to declare.

## ETHICS STATEMENT

This study was conducted at an academic hospital complex affiliated with Tehran University of Medical Sciences (TUMS), Tehran, Iran, and approved by the ethics committee of the university. Patient's anonymity and confidentiality were carefully protected, according to the Declaration of Helsinki.

## CONSENT

Written informed consent was obtained from the patient to publish this report in accordance with the journal's patient consent policy.

## Supporting information


Data S1:

## Data Availability

Data sharing does not apply to this article as no datasets were generated or analyzed during this study.
