# Supplementary material for: Coexistence of myasthenia gravis and lichen planus: A case report and systematic review of related case reports from 1971 to 2024
Source: Clin Case Rep. 2024 Jun 14;12(6):e9065. doi: 10.1002/ccr3.9065 (PMC11177179; doi:10.1002/ccr3.9065)
Supplement: Supplementary file 1 — Data S1: [file CCR3-12-e9065-s001.docx]

# Supplementary Materials

**Coexistence of myasthenia gravis and lichen planus: A case report and systematic review of related case reports from 1971 to 2024**

| **Contents** |  | **Page** |
| --- | --- | --- |
| **Table S1** | **The CARE Guidelines: Consensus-based Clinical Case Reporting Guideline Development** | [**1**](#_Table_S1._The) |
| **Table S2** | **Systematic search syntax for MEDLINE (via PubMed), Scopus, and Web of Sciences with results as of February 01, 2024** | [**2**](#_Table_S2._Systematic) |

# **
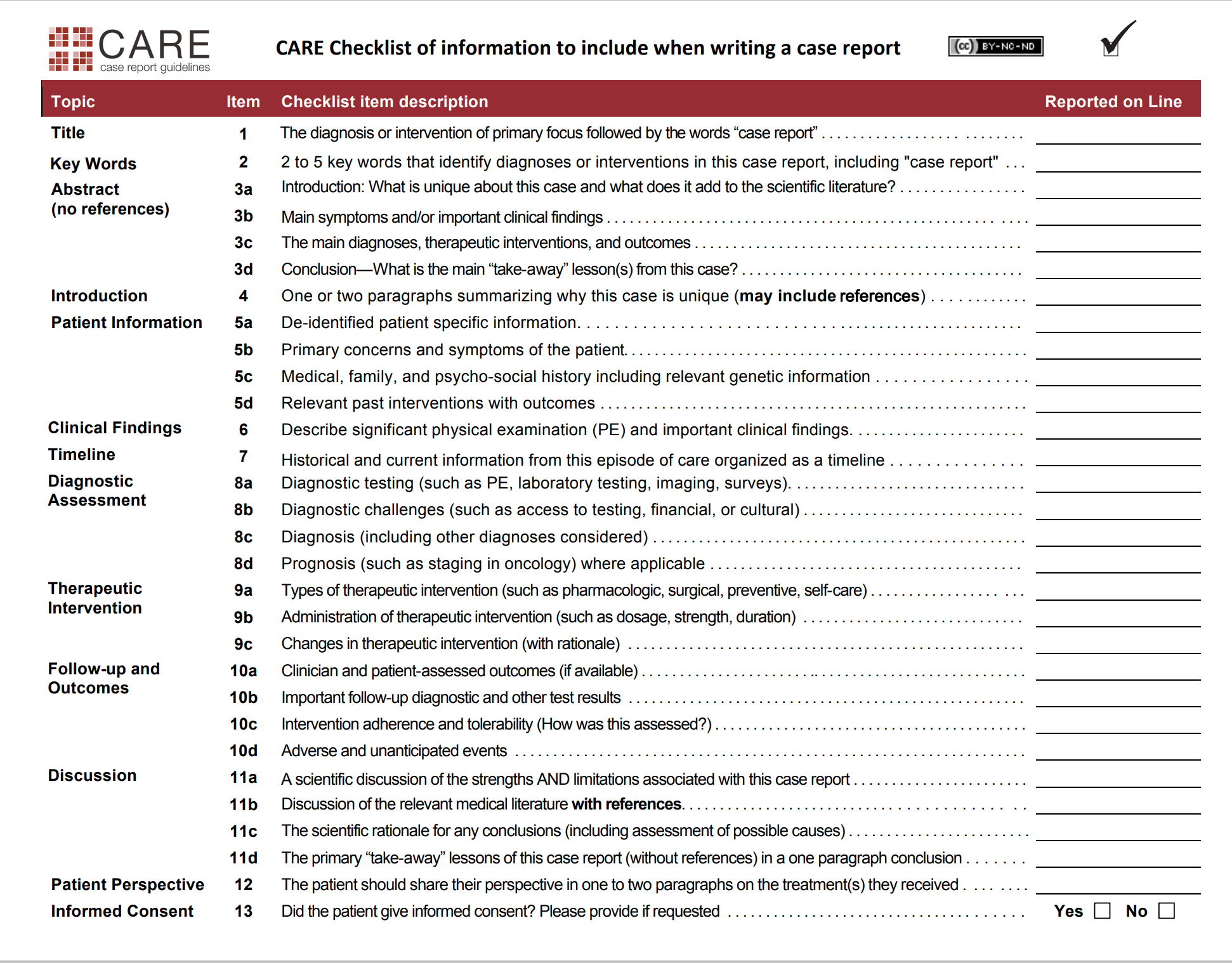
Table S1. The CARE Guidelines: Consensus-based Clinical Case Reporting Guideline Development**

NA

13

14, KCM

14

10-13

8

NA

NA

8

NA

8

8

8

NA

NA

8

7, Table 1

7

7

7

7

7

3-4

2

2

2

2

2

0

# **Table S2. Systematic search syntax for MEDLINE (via PubMed), Scopus, and Web of Sciences with results as of February 01, 2024**

| **No.** | **Syntax** | **No. of results** |
| --- | --- | --- |
| **PubMed** | (“Myasthenia Gravis”[Mesh] OR “myasthenia gravis”[Title/Abstract] OR myasthenia[Title/Abstract] OR myasthenic [Title/Abstract]) AND ("Lichen Planus"[Mesh] OR “lichen planus” [Title/Abstract] OR lichenoid [Title/Abstract]) | [31](https://pubmed.ncbi.nlm.nih.gov/?term=%28%E2%80%9CMyasthenia+Gravis%E2%80%9D%5BMesh%5D+OR+%E2%80%9Cmyasthenia+gravis%E2%80%9D%5BTitle%2FAbstract%5D+OR+myasthenia%5BTitle%2FAbstract%5D+OR+myasthenic+%5BTitle%2FAbstract%5D%29+AND+%28%22Lichen+Planus%22%5BMesh%5D+OR+%E2%80%9Clichen+planus%E2%80%9D+%5BTitle%2FAbstract%5D+OR+lichenoid+%5BTitle%2FAbstract%5D%29&sort=fauth&ac=no) |
| **Scopus** | (TITLE-ABS-KEY (“myasthenia gravis”) OR TITLE-ABS-KEY (myasthenia) OR TITLE-ABS-KEY ( myasthenic )) AND (TITLE-ABS-KEY ( “lichen planus”) OR TITLE-ABS-KEY ( lichenoid)) | **123** |
| **Web of Science** | (TS=(“myasthenia gravis”) OR TS=( myasthenia) OR TS=( myasthenic)) AND (TS=( “lichen planus”) OR TS=( lichenoid)) | **42** |
| **Total** |  | **196** |
